# O060: Reduction of central-line associated bloodstream infections in a tertiary care hospital in Saudi Arabia

**DOI:** 10.1186/2047-2994-2-S1-O60

**Published:** 2013-06-20

**Authors:** WA Mazi, Z Bejum, D Abdulmutalib, A Hisham, S Maghari, M Al Thumali, A Senok

**Affiliations:** 1 infection prevention and control, Saudi Arabia; 2 Intensive Care Unit, King Abdul Aziz Specialist Hospital, Taif, Saudi Arabia; 3 College of Medicine, Alfaisal University, Riyadh, Saudi Arabia

## Introduction

Central line-associated bloodstream infection (CLABSI) remains a major problem in critical care units worldwide.

## Objectives

This study aimed to assess the impact of the implementation the Society for Healthcare Epidemiology of America/ Infectious Diseases Society of America (SHEA/IDSA) practice recommendations in reducing CLABSI rates in an acute trauma intensive care unit (ICU).

## Methods

The prospective study was conducted from January 2011-December 2012 at the 23-bed trauma-ICU in King Abdul Aziz Specialist Hospital, Taif, Saudi Arabia. In 2011, baseline data on CLABSI rates was collected. In 2012, a CLABSI-Team was established and the basic SHEA/IDSA practice recommendations implemented. Laboratory-confirmed CLABSI were identified using the Centers for Disease Control and Prevention criteria and antimicrobial susceptibility of isolates determined. For benchmarking with National Healthcare Safety Network (NHSN, USA), data collection and analysis were carried out in accordance with NHSN recommendations.

## Results

With implementation of the SHEA/IDSA practice recommendations, a decline in the number of CLABSI in 2012 (n=6) compared to 2011 (n=14) was observed. This corresponds to a 58% decline in CLABSI incidence rate from 3.87 to 1.5 per 1000 central-line days in 2011 and 2012 respectively (Standardized Infection Ratio, 0.42). Benchmarking to NHSN percentiles, the incidence of CLABSI was 75^th^-90^th^ percentile in 2011 *vs.* 50^th^ percentile in 2012. The utilization ratio was 25^th^-50^th^ percentile in 2011 and 50^th^-75^th^ percentile in 2012. Three *Klebsiella pneumoniae* isolates susceptible only to imipenem and one pan-drug resistant *Acinetobacter baumanii* were identified in 2012. Two patients had *Enterococcus faecalis*, with one isolate resistant to vancomycin.

## Conclusion

Implementation of the basic SHEA/IDSA practice recommendations resulted in significant reduction in CLABSI in our trauma-ICU where multidrug resistant isolates are present indicative of the beneficial role of these recommendations.

## Disclosure of interest

None declared.

